# Comparative Metagenomic Analysis of Rhizosphere Microbial Community Composition and Functional Potentials under *Rehmannia glutinosa* Consecutive Monoculture

**DOI:** 10.3390/ijms19082394

**Published:** 2018-08-14

**Authors:** Linkun Wu, Juanying Wang, Hongmiao Wu, Jun Chen, Zhigang Xiao, Xianjin Qin, Zhongyi Zhang, Wenxiong Lin

**Affiliations:** 1 College of Life Sciences, Fujian Agriculture and Forestry University, Fuzhou 350002, China; wulinkun619@163.com (L.W.); juanying020@163.com (J.W.); wuhongmiao2010@163.com (H.W.); chenjunfafu@163.com (J.C.); m18305987298@163.com (Z.X.); 2 Fujian Provincial Key Laboratory of Agroecological Processing and Safety Monitoring, Fujian Agriculture and Forestry University, Fuzhou 350002, China; xianjinqin1026@163.com; 3 Key Laboratory of Crop Ecology and Molecular Physiology (Fujian Agriculture and Forestry University), Fujian Province University, Fuzhou 350002, China; zyzhang@fafu.edu.cn; 4 College of Crop Science, Fujian Agriculture and Forestry University, Fuzhou 350002, China

**Keywords:** *Rehmannia glutinosa*, soil sickness, metagenomics, rhizosphere microbiome, functional potential

## Abstract

Consecutive monoculture of *Rehmannia glutinosa*, highly valued in traditional Chinese medicine, leads to a severe decline in both quality and yield. Rhizosphere microbiome was reported to be closely associated with the soil health and plant performance. In this study, comparative metagenomics was applied to investigate the shifts in rhizosphere microbial structures and functional potentials under consecutive monoculture. The results showed *R. glutinosa* monoculture significantly decreased the relative abundances of *Pseudomonadaceae* and *Burkholderiaceae*, but significantly increased the relative abundances of *Sphingomonadaceae* and *Streptomycetaceae*. Moreover, the abundances of genera *Pseudomonas*, *Azotobacter*, *Burkholderia*, and *Lysobacter*, among others, were significantly lower in two-year monocultured soil than in one-year cultured soil. For potentially harmful/indicator microorganisms, the percentages of reads categorized to defense mechanisms (i.e., ATP-binding cassette (ABC) transporters, efflux transporter, antibiotic resistance) and biological metabolism (i.e., lipid transport and metabolism, secondary metabolites biosynthesis, transport and catabolism, nucleotide transport and metabolism, transcription) were significantly higher in two-year monocultured soil than in one-year cultured soil, but the opposite was true for potentially beneficial microorganisms, which might disrupt the equilibrium between beneficial and harmful microbes. Collectively, our results provide important insights into the shifts in genomic diversity and functional potentials of rhizosphere microbiome in response to *R. glutinosa* consecutive monoculture.

## 1. Introduction

**Figure 1:**
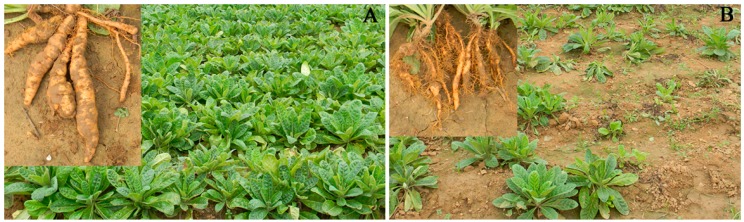
Photographs of above and below ground components of *R. glutinosa* under one-year (**A**) and two-year (**B**) consecutive monoculture.

The term allelopathy generally describes chemical interactions between different plant species due to the biochemicals that they release into the environment [1]. Allelopathic autotoxicity, a special allelopathy phenomenon, refers to the harmful effect that one plant species causes to itself as a result of repeated planting of the same plant species in the same soil for multiple years [2]. Allelopathic autotoxicity, also known as the consecutive monoculture problem or “soil sickness”, commonly occurs in the monoculture cultivation of a range of crops in intensive agriculture, including food crops (such as wheat and potato), vegetables (such as cucumber and tomato), and medicinal plants (such as *Rehmannia glutinosa* and *Panax ginseng*) [3,4]. Numerous medicinal plants suffer from a serious consecutive monoculture problem. Approximately 70% of medicinal plant species with tuberous roots have various degrees of consecutive monoculture problems [4,5], which substantially limits the development of traditional Chinese medicines. *R. glutinosa*, belonging to the family *Scrophulariaceae*, is highly valued in traditional Chinese medicine for curing endocrine, immune, cardiovascular, and nervous system ailments [6,7]. It is mainly produced in Jiaozuo city, Henan Province, central China, which is recognized as the geo-authentic production zone with the most favourable soil and climatic conditions [8]. However, consecutive monoculture of *R. glutinosa* has resulted in a serious decline in both yield and quality of tuberous roots (Figure 1). In addition, consecutive monoculture of *R. glutinosa* significantly increased the abundances of *Fusarium oxysporum* and *Aspergillus flavus* in rhizosphere, two main causal agents of *R. glutinosa* root rot diseases [7,9]. Fields used for *R. glutinosa* cultivation can only be replanted once in every 15–20 years [10,11]. Besides, a relatively large quantity of pesticide and fertilizer was applied by farmers to maintain high production in a monoculture regime. However, it elevates agricultural inputs, and causes excessive chemical residues on the crop and environment damage. It has, therefore, become urgent to understand the underlying mechanisms of consecutive monoculture problems exhibited in *R. glutinosa*.

Previous research into consecutive monoculture problems has mainly focused on the deficiency in soil nutrients and direct inhibitory effect (direct allelopathy) of an exuded metabolite on the recipient plant using filter paper or agar bioassays enriched with a single chemical [12,13]. However, our previous study has indicated that soil organic matter and most available nutrients did not decrease under *R. glutinosa* consecutive monoculture [14]. Such evidence for direct allelopathy is generally insufficient as the concentrations required for growth inhibition are generally higher than those found in natural conditions, and bioassays do not represent the degradation and transformation of these chemicals by the soil microbial community [12]. By contrast, Li et al. [15] demonstrated that the consecutive monoculture problems in peanut were attributable to the changes in the soil microbial community structure induced by root exudates (indirect allelopathy) rather than the direct allelopathy. Xiong et al. [16] found that long-term consecutive monoculture of black pepper (*Piper nigrum* L.) resulted in a decrease in soil bacterial abundance and altered soil microbial community membership and structure. Our previous studies using terminal restriction fragment length polymorphism (T-RFLP) and phospholipid fatty acid (PLFA) profiles analysis also found that *R. glutinosa* monoculture could alter both soil bacterial and fungal communities [7,14]. Therefore, a growing body of evidence suggests that plant–microbe interactions play crucial roles in soil quality and crop health [4,17,18,19].

Using Illumina sequencing of small subunit ribosomal RNA (SSU rRNA) genes, previous studies demonstrated that consecutive monoculture of *R. glutinosa* altered the microbial community structure in rhizosphere soil, and led to the imbalance of the microbial community [8,20]. However, there exists limited knowledge of the functional attributes of root-associated microbes in the rhizosphere soil of *R. glutinosa*, and the linkages between the variation of functional genes and the dynamics of microbial community. Moreover, the 16S rRNA gene is highly conserved in regard to sequence similarity; hence, even species with identical or almost identical 16S rRNA genes can have very different functional and/or ecological differentiation [21]. In recent years, comparative metagenomics on the whole-genome has proven to be a powerful tool to analyze community-wide shifts in response to environmental perturbations and link the functional genes of uncultured organisms to phylogenetic groups [21,22]. Unfortunately, however, little is known about the changes in the microbial community structure and functional potentials in *R. glutinosa* rhizosphere under a monoculture regime.

Therefore, it is necessary to perform whole-community shotgun metagenomic sequencing to better understand the genomic diversity and functional attributes of complex soil microbial communities. In the current study, we examined the shifts in composition and predicted metabolism of rhizospheric microbial communities under *R. glutinosa* monoculture based on whole-genome shotgun sequence analysis.

## 2. Results

### 2.1. The Morphology of R. glutinosa under Consecutive Monoculture

Compared with the one-year cultured (NP) plants (Figure 1A), the two-year consecutively monocultured (CM) plants displayed poorer growth as indicated by the decreased below-ground biomass and large numbers of adventitious fibrous roots (Figure 1B). The fresh tuber weight was significantly (*p* < 0.01) higher in the one-year planted plots (79.7 g per plant, standard deviation (SD) = 7.47, *n* = 5) than in the two-year monocultured plots (27.2 g per plant, SD = 6.71, *n* = 5). Moreover, approximately 40% of consecutively-monocultured plants were found to suffer from severe wilt disease and died off (Figure 1).

### 2.2. Overview of Metagenomic Sequencing, Assembly, and Annotation

A total of 37,712 Mbp clean reads were generated from the metagenomic libraries (three replicated libraries from NP, denoted as NP1, NP2, and NP3; three replicated libraries from CM, denoted as CM1, CM2, and CM3) and the ratio of clean data to raw data was an average of 97.35%. Sequence assembly using the SOAPdenovo software generated 14,014, 12,426, and 11,875, and 11,865, 16,488, and 15,344 scaftigs longer than 500 bp in NP1–NP3 and CM1–CM3, respectively. On average, the scaftigs N50 lengths were 844 and 813 bp in NP and CM, respectively, and scaftigs N90 lengths were 540 and 539 bp in NP and CM, respectively. Gene prediction using MetaGeneMark software showed that there were 71,246 non-redundant open reading frames (ORFs) yielded from the metagenomic libraries and 15,672 (22%) of them were complete ORFs with both initiation codon and termination codon (Table S1). Rarefaction curves generated for the species level were approaching plateaus (Figure S1), suggesting that all libraries represented the microbial communities well. Furthermore, phylogenetic analysis using DIAMOND software showed that 58,760 (82.47%) of the predicted ORFs could be annotated on National Center for Biotechnology Information (NCBI) microbial non-redundant (microNR) database. In total, we were able to classify approximately 93.54%, 91.87%, 89.76%, 87.71%, 86.60%, 73.95%, and 53.95% of the predicted ORFs at the kingdom, phylum, class, order, family, genus, and species levels, respectively. Functional annotation showed that 52,943 (74.31%) and 50,245 (70.52%) of the predicted ORFs could be annotated on Kyoto Encyclopedia of Genes and Genomes (KEGG) and Evolutionary genealogy of genes: Non-supervised Orthologous Groups (eggNOG) databases, respectively. Among them, 30,962 (43.46%) of the predicted ORFs could be assigned to 2985 KEGG ortholog (KO) groups on KEGG ORTHOLOGY database, and 18,289 (25.67%) of the predicted ORFs could be assigned to 285 pathways on KEGG PATHWAY database. Besides, 50,245 (70.52%) of the predicted ORFs could be assigned to 6325 orthologous groups (OGs) on eggNOG database.

### 2.3. Core/Pan and Venn Diagram Analyses

Core/pan analysis showed that the number of genes shared among six soil samples was 36,979, with a low number of genes exclusively found in individual soil samples (Figure S2A). Venn diagram indicated that the number of genes shared in NP and CM was 54,073, and the numbers of genes exclusively found in NP and CM were 4508 and 4402, respectively (Figure S2B). Furthermore, correlation analysis among different samples revealed distinct differences between two treatments (NP and CM) and highly similar patterns for the three replicates in each treatment (Figure S2C).

### 2.4. Phylogenetic Analysis of Rhizosphere Microbial Communities under Consecutive Monoculture

**Figure 2:**
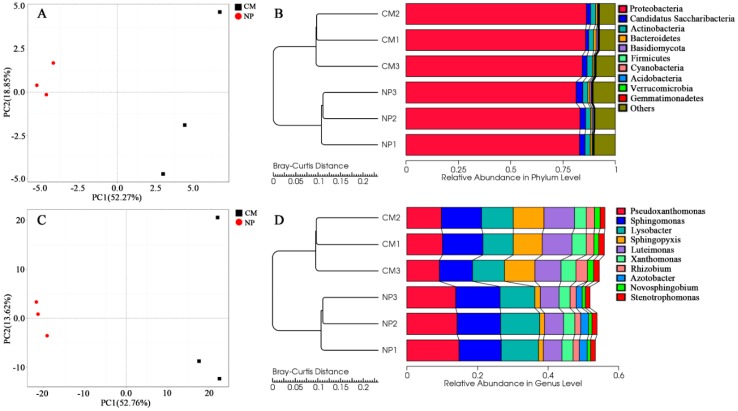
Classification of treatments by principal component analysis (PCA) and unweighted pair-group method with arithmetic mean (UPGMA) clustering analyses at phylum level (**A**,**B**) and genus level (**C**,**D**). NP and CM represent the one-year cultured soil and two-year consecutively monocultured soil, respectively.

Principal component analysis (PCA) at both phylum level and genus level showed distinct differences in microbial communities between NP and CM. The first two principal components (PC1 and PC2) of PCA at phylum level explained 52.27% and 18.85% of the total variations, respectively and explained 52.76% and 13.62%, respectively, at genus level (Figure 2A,C). Unweighted pair-group method with arithmetic mean (UPGMA) clustering based on Bray–Curtis distance revealed a similar microbial community structure for the three replicates in each treatment and obvious differences between two treatments (NP and CM) (Figure 2B,D).

Phylogenetic analysis showed that 92.64%, 0.05%, and 0.48% of the predicted ORFs could be assigned to kingdoms bacteria, archaea and eukaryota, respectively. The microbial community was comprised mainly of ten phyla, *Proteobacteria*, *Candidatus Saccharibacteria*, *Actinobacteria*, *Bacteroidetes*, *Basidiomycota*, *Firmicutes*, *Cyanobacteria*, *Acidobacteria*, *Verrucomicrobia*, and *Gemmatimonadetes* (Figure 2B,D). *Proteobacteria* was the dominant microbial taxa, accounting for 82.4% and 85.4% of the total population in NP and CM, respectively. At the family level, *R. glutinosa* consecutive monoculture significantly (*q* < 0.05) increased the relative abundances of *Sphingomonadaceae* and *Streptomycetaceae*, but significantly (*q* < 0.05) decreased the relative abundances of *Pseudomonadaceae*, *Burkholderiaceae*, *Rhodobacteraceae*, and *Enterobacteriaceae*, among others. (Figure S3).

**Figure 3:**
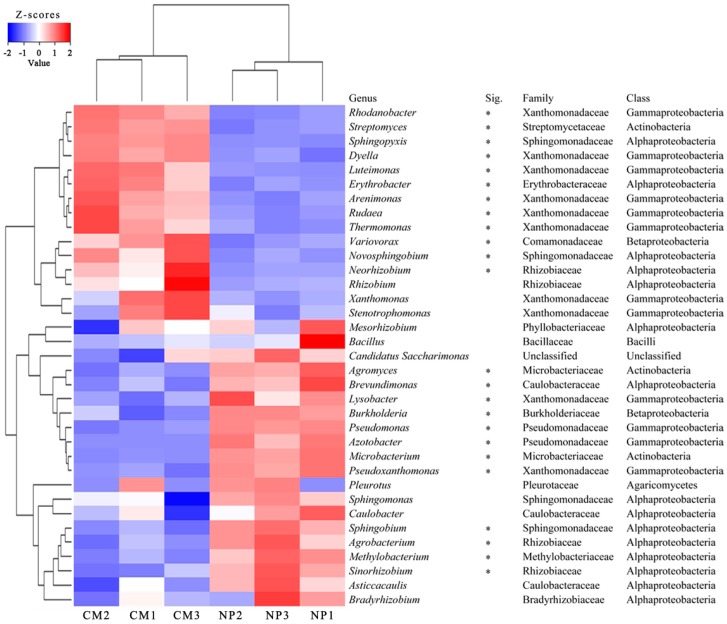
Heat map analysis of the top 35 most abundant genera in at least one soil sample using normalized abundance. Heat map is color-coded based on row z-scores. NP and CM represent the one-year cultured soil and two-year consecutively monocultured soil, respectively. Sig. represents significant differences for corresponding genera between two different treatments (* *q* < 0.05).

Furthermore, at the genus level, 808 taxa (including prokaryotic and eukaryotic organisms) were classified from NP1–NP3 and CM1–CM3. The top 35 most abundant genera in at least one soil sample accounted for 64.26% and 65.64% of the total population in NP and CM, respectively. A heat map visualization with hierarchical clustering of the top 35 most abundant genera in at least one soil sample showed distinct differences in microbial community structure between NP and CM (Figure 3). Moreover, most predominant genera were significantly different between NP and CM. Specifically, the relative abundances of *Sphingopyxis* and *Novosphingobium* (belonging to *Sphingomonadaceae*); *Streptomyces* (belonging to *Streptomycetaceae*); and *Luteimonas*, *Arenimonas*, and *Rhodanobacter* (belonging to *Xanthomonadaceae*) were significantly (*q* < 0.05) higher in CM than in NP, but the opposite was true for the relative abundances of *Lysobacter* and *Pseudoxanthomonas* (belonging to *Xanthomonadaceae*), *Pseudomonas* and *Azotobacter* (belonging to *Pseudomonadaceae*), and *Burkholderia* (belonging to *Burkholderiaceae*), among others. (Figure 3).

### 2.5. Kyoto Encyclopedia of Genes and Genomes (KEGG) Analysis

The genes assigned to metabolism were dominant among the categories, especially for carbohydrate metabolism and amino acid metabolism, and followed by genetic information processing and environmental information processing (Figure S4). Besides, nucleotide metabolism and translation were significantly (*q* < 0.05) higher in NP than in CM, but the opposite was true for the lipid metabolism (Figure S4).

**Figure 4:**
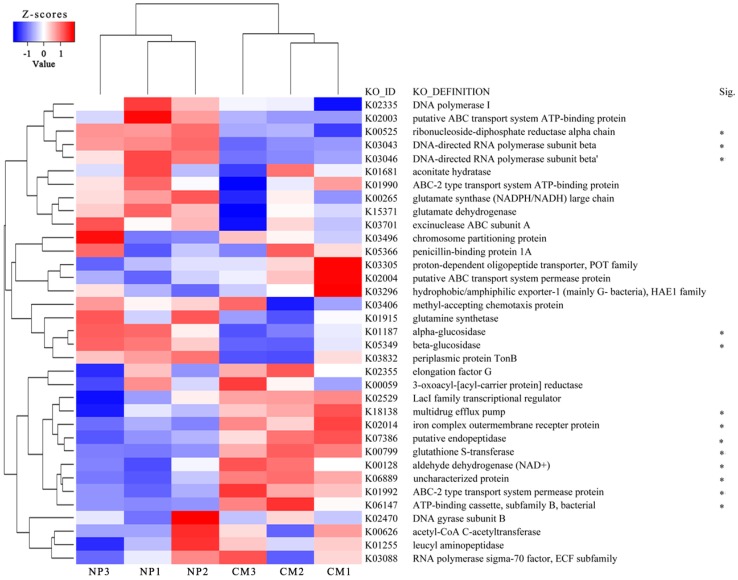
Heat map analysis of the top 35 most abundant Kyoto Encyclopedia of Genes and Genomes (KEGG) ortholog (KO) groups in at least one soil sample using normalized abundance. Heat map is color-coded based on row z-scores. Abbreviations ATP, NADPH/NADH and ECF represent adenosine triphosphate, nicotinamide adenine dinucleotide phosphate/nicotinamide adenine dinucleotide and extracytoplasmic function, respectively. NP and CM represent the one-year cultured soil and two-year consecutively monocultured soil, respectively. Sig. represents significant differences for KOs between two different treatments (* *q* < 0.1).

PCA analysis based on the relative abundances of KOs showed distinct differences in functional potentials of microbial communities between NP and CM. PC1 and PC2 explained 43.01% and 15.45% of the total variations, respectively (Figure S5). The heat map of the top 35 most abundant KOs in at least one soil sample also showed distinct differences between NP and CM (Figure 4). The percentages of reads categorized to ribonucleoside–diphosphate reductase alpha chain (K00525, nucleotide metabolism), DNA-directed RNA polymerase subunit beta (K03043 and K03046, nucleotide metabolism), alpha-glucosidase (K01187, carbohydrate metabolism), and beta-glucosidase (K05349, carbohydrate metabolism) were significantly (*q* < 0.1) higher in NP than in CM. However, the clusters of ABC-2 type transport system permease protein (K01992, ABC transporters), ATP-binding cassette subfamily B (K06147, ABC transporters), iron complex outermembrane recepter protein (K02014, transporters), multidrug efflux pump (K18138, drug efflux transporter), glutathione *S*-transferase (K00799, metabolism of other amino acids, xenobiotics biodegradation and metabolism), and aldehyde dehydrogenase (NAD^+^) (K00128, lipid metabolism and carbohydrate metabolism), among others, were significantly (*q* < 0.1) lower in NP than in CM (Figure 4). Furthermore, it was found that the relatively higher abundances of K00525 and K03043 in NP were attributed to the higher abundances of these KOs derived from *Lysobacter*, *Pseudoxanthomonas*, *Sphingomonas*, *Thermomonas*, and *Azotobacter* in NP (Figure S6). The relatively higher abundances of K01992 and K06147 in CM were mainly attributed to the higher abundances of these KOs derived from *Sphingopyxis*, *Luteimonas*, *Arenimonas*, and *Xanthomonas* in CM (Figure S6). The relatively higher abundances of K18138 and K00799 in CM were mainly attributed to the higher abundances of these KOs derived from *Sphingopyxis* and *Luteimonas* in CM (Figure S6).

**Figure 5:**
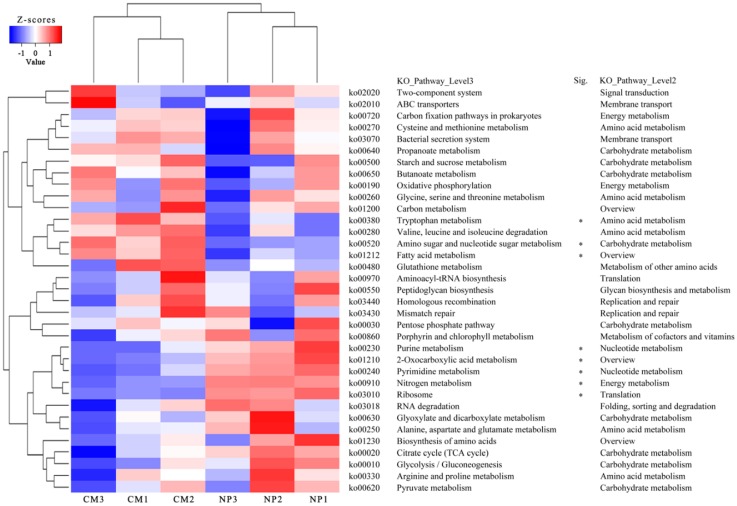
Heat map analysis of the top 35 most abundant KEGG pathways in at least one soil sample using normalized abundance. Heat map is color-coded based on row z-scores. The metabolic pathways assigned to “Organismal Systems” and “Human Diseases” on KEGG database were excluded. NP and CM represent the one-year cultured soil and two-year consecutively monocultured soil, respectively. Sig. represents significant differences for level 3 KEGG pathways between two different treatments (* *q* < 0.12).

The vast majority of biochemical reactions in the KEGG metabolic pathways were detected in both NP and CM, as indicated by red lines implying that they shared similar bio-information (Figure S7). However, heat map analysis of the top 35 most abundant KEGG pathways in at least one soil sample showed distinct differences between NP and CM (Figure 5). The percentages of reads categorized to purine metabolism (ko00230, nucleotide metabolism), pyrimidine metabolism (ko00240, nucleotide metabolism), ribosome (ko03010, translation), nitrogen metabolism (ko00910, energy metabolism), and 2-oxocarboxylic acid metabolism (ko01210, overview) were significantly (*q* < 0.12) higher in NP than in CM (Figure 5), which was mainly because of the higher abundances of these pathways derived from *Sphingomonas*, *Lysobacter*, *Pseudoxanthomonas*, and *Azotobacter* in NP (Figure S8). However, the percentages of reads categorized to fatty acid metabolism (ko01212, overview), tryptophan metabolism (ko00380, amino acid metabolism), amino sugar, and nucleotide sugar metabolism (ko00520, carbohydrate metabolism) were significantly (*q* < 0.12) higher in CM than in NP (Figure 5). Furthermore, heat map analysis of all differential (*q* < 0.12) KEGG pathways showed that the percentages of reads categorized to beta-lactam resistance (ko01501, drug resistance), penicillin and cephalosporin biosynthesis (ko00311), replication and repair (ko03410, ko03450), and xenobiotics biodegradation and metabolism (ko00980, ko00982) were significantly higher in CM than in NP (Figure S9). The relatively higher abundances of ko01212, ko01501, ko00311, ko03410, ko03450, ko00980, and ko00982 in CM were mainly attributed to the higher abundances of these KEGG pathways derived from *Sphingopyxis*, *Luteimonas*, *Novosphingobium*, and *Xanthomonas* in CM (Figure S10).

### 2.6. eggNOG Analysis

The clusters of [F] nucleotide transport and metabolism and [N] cell motility were significantly higher in NP than in CM (Supplementary Figure S11), mainly because of the higher abundances of these clusters derived from *Pseudoxanthomonas*, *Sphingomonas*, *Lysobacter*, and *Azotobacter* in NP (Figure S12). However, the clusters of [I] lipid transport and metabolism; [Q] secondary metabolites biosynthesis, transport, and catabolism; [V] defense mechanisms; [P] inorganic ion transport and metabolism; and [K] transcription were significantly higher in CM than in NP (Figure S11), mainly because of the higher abundances of these clusters derived from *Sphingopyxis*, *Luteimonas*, *Xanthomonas*, and *Novosphingobium* in CM (Figure S12).

The heat map of the top 35 most abundant OGs in at least one soil sample showed that Cluster of Orthologous Groups of proteins (COG) categories COG1960 ([I] lipid transport and metabolism), COG1680 ([V] defense mechanisms), COG0841, COG1629 and ENOG410XQKN ([P] inorganic ion transport and metabolism), COG1595 ([K] transcription), COG1506 ([E] amino acid transport and metabolism), and COG2885 ([M] cell wall/membrane/envelope biogenesis) were significantly higher in CM than in NP (Figure S13). However, COG4638 ([P] inorganic ion transport and metabolism) and COG0494 ([L] replication, recombination, and repair) were significantly higher in NP than in CM (Figure S13).

### 2.7. Function Potential Analysis of Specific Microbes

Genera *Lysobacter*, *Pseudomonas*, *Azotobacter*, *Sphingomonas*, *Sphingopyxis*, and *Luteimonas* were predominant in all soil samples and most of them showed significant differences in relative abundances between NP and CM. Therefore, the changes in the function potentials associated with these genera were evaluated based on the metagenomic sequencing using COG analysis. The function potentials that were more abundant in NP than in CM, identified from *Lysobacter*, *Pseudomonas*, *Azotobacter*, and *Sphingomonas* assembly, included [I] lipid transport and metabolism; [F] nucleotide transport and metabolism; [K] transcription; [J] translation, ribosomal structure, and biogenesis; [E] amino acid transport and metabolism; [G] carbohydrate transport and metabolis; [C] energy production and conversion; [H] coenzyme transport and metabolism; [N] cell motility; [M] cell wall/membrane/envelope biogenesis; [T] signal transduction mechanisms; and [D] cell cycle control, cell division, and chromosome partitioning (Figure S14). In comparison, in addition to the COG categories mentioned above, others including [L] replication, recombination, and repair; [V] defense mechanisms; [P] inorganic ion transport and metabolism; [Q] secondary metabolites biosynthesis, transport, and catabolism; [O] posttranslational modification, protein turnover, and chaperones; and [U] intracellular trafficking, secretion, and vesicular transport identified from *Sphingopyxis* and *Luteimonas* assembly were significantly higher in CM than in NP (Figure S14).

## 3. Discussion

Rhizosphere is the narrow region of soil that is directly influenced by living roots, and the primary site of interaction between plants and microorganisms [23,24]. Many studies demonstrated that rhizosphere microbial community can be shaped by plant host habitat, root exudates, and root architectural or phenotypic traits [25,26,27]. The collective genome of rhizosphere microbial community is referred to as the “second genome” of the plant, which is vital for plant health [23,28]. An increasing number of research studies have shown that indirect allelopathy through modifications of the rhizosphere microbial community by the biochemicals is closely related to the monoculture problems in agriculture and horticulture [8,15,29,30]. Our previous studies via barcoded pyrosequencing of 16S rRNA genes and internal transcribed spacer (ITS2) amplification have revealed the shifts in the bacterial and fungal community diversity under *R. glutinosa* consecutive monoculture [20,31]. However, limited information was available about how the functional traits of soil microbial communities vary under *R. glutinosa* monoculture.

In this study, the PCA and cluster analysis based on metagenomic sequencing data showed a discernible separation in rhizosphere microbial community structure among the one-year cultured soil and the two-year monocultured soil (Figure 2). However, further work is need to distinguish the changes in soil microbial community caused by the different physiologies or phenotypes of the plants under *R. glutinosa* monoculture and to evaluate the importance of root exudates or preceding residues in determining rhizosphere bacterial community structure. In the current work, it was found that *R. glutinosa* monoculture resulted in a significant decrease in the relative abundances of *Pseudomonadaceae* and *Burkholderiaceae*, among others, at the family level, and a significant reduction in the relative abundances of *Pseudomonas* and *Azotobacter* (belonging to *Pseudomonadaceae*), *Burkholderia* (belonging to *Burkholderiaceae*), and *Lysobacter* (belonging to *Xanthomonadaceae*), among others, at the genus level. The opposite was true for *Sphingomonadaceae* and *Streptomycetaceae* (Figure 3 and Figure S3). This is in line with our previous study through 16S rRNA amplicon pyrosequencing where the relative abundances of *Pseudomonadaceae* and *Pseudomonas* decreased while *Sphingomonadaceae* increased under consecutive monoculture [8]. Many previous findings have demonstrated the concomitant occurrence of *Sphingomonadaceae* and *Streptomycetaceae* in the rhizosphere under consecutive monoculture or upon fungal pathogen invasion [32,33]. Members of *Streptomycetaceae* and *Sphingomonadaceae* living in soil could decompose dead plants and fungi by synthesizing cellulose-, pectin-, xylan-, and chitin-degrading enzymes [32,34,35]. Therefore, the increase of *Sphingomonadaceae* and *Streptomycetaceae* in rhizosphere might be important indicator microorganisms of root damage by soil fungal pathogens [32], but more direct and comprehensive studies are required to test this hypothesis in *R. glutinosa* monoculture regime. In contrast, high abundances of several bacterial taxa including *Pseudomonadaceae*, *Burkholderiaceae*, and *Xanthomonadales* in sugar beet rhizosphere have been proposed to contribute to the soil disease suppressiveness [36]. In our previous study, we also demonstrated the strong antagonistic activities of many members of genus *Pseudomonas* towards soil-borne pathogens *F. oxysporum* or *A. flavus*, two main agents known to cause wilt and rot disease of *R. glutinosa* [7,8,9]. Therefore, the declined soil suppressiveness to fungal pathogens in *R. glutinosa* monoculture regime might be associated with the reduction in the abundances of these antagonistic microbes in rhizosphere. However, it is should be noted that limited information was available about the fungal community in this study because phylogenetic information was deduced from the best hits obtained when annotating ORFs. However, databases are largely dominated by annotated genes from cultivable bacteria belonging to proteobacterial classes. Additionally, it is reported that shotgun sequencing could underestimate the abundance of fungal sequences [37]. Further work by other techniques such as metatranscriptomics targeting polyA RNA [38] is needed to study the root-associated fungal community structure and functions under *R. glutinosa* consecutive monoculture.

The whole-community shotgun metagenomics sequencing has proven to be a powerful tool to characterize the metabolic potentials of the soil microbial community, and link the functional genes of uncultured organisms to phylogenetic groups. It should be noted, however, that microbial gene expression in the rhizosphere cannot be captured by metagenomics approach, but only indirectly inferred. In this study, the results based on the metagenomics data showed that the percentages of reads categorized to nucleotide metabolism and translation were significantly higher in NP than in CM, but the opposite was true for the lipid metabolism (especially fatty acid metabolism) (Figure 5 and Figure S4). Besides, it was found that the percentages of reads categorized to biological metabolism (lipid transport and metabolism, secondary metabolites biosynthesis, transport and catabolism, nucleotide transport and metabolism), genetic information processing (transcription, translation, DNA repair, etc.), signal transduction, defense mechanisms (ABC transporters, efflux transporter, antibiotic resistance), and cell wall/membrane/envelope biogenesis were significantly higher in NP than in CM for beneficial microorganisms (i.e., *Lysobacter*, *Pseudomonas*, *Azotobacter*, etc.), but the opposite was true for harmful/indicator microorganisms (i.e., *Sphingopyxis*, *Novosphingobium*, *Streptomyces*, etc.) (Figures S10, S12 and S14). Among them, the relatively higher abundance of fatty acid metabolism (ko01212) or [I] lipid transport and metabolism in CM was mainly attributed to the higher abundance of this feature derived from *Sphingopyxis*, *Luteimonas*, *Novosphingobium*, and *Xanthomonas* in CM (Figures S10 and S12). Fatty acids are essential components of microbial membranes and are important for environmental stresses response and survival. Fozo and Quivey [39] demonstrated that an increased proportion of long-chained, monounsaturated fatty acids in membranes is important for survival in acidic environments. Indeed, our previous study indicated that soil pH significantly decreased under *R. glutinosa* consecutive monoculture [14]. Therefore, the higher abundance of fatty acid metabolism derived from these potentially harmful or indicator microbes in CM might contribute to the acid survival for them in the monocultured soil. Additionally, Li et al. [40] demonstrated that the up-regulation of fatty acid biosynthesis was crucial for the biofilm fitness and antibiotic resistance in bacterial pathogens.

Furthermore, the metagenomics analysis showed that the levels of the clusters of defense mechanisms (i.e., antibiotic resistance, ABC transporters, efflux transporter) and DNA repair were higher in CM than in NP for harmful/indicator microorganisms (Figures S12 and S14), which might confer them high tolerance levels to toxic or antimicrobial compounds [41,42]. Various studies indicated that consecutive monoculture of medicinal plants led to the accumulation of toxic compounds in soil, such as microbial secondary metabolites, autotoxins released by plant roots, and so on [43,44,45]. In this study, it was found that the percentage of reads categorized to penicillin and cephalosporin biosynthesis (ko00311) was significantly higher in CM than in NP (Figure S9). It might also reflect elevated microbial competition in the consecutively-monocultured soils. Fierer et al. [46] suggested that elevated microbe–microbe competition should select for increased antibiotic production and resistance. In addition, our previous studies showed that *R. glutinosa* could secrete many phenolic compounds with antimicrobial activity in root exudates, and hence selectively stimulate and/or inhibit various microbes [7,47]. In detail, a mixture of phenolic acids that were identified in the root exudates of *R. glutinosa* could accelerate the mycelial growth and mycotoxin production of pathogenic *F. oxysporum*, but suppress the growth of the beneficial *Pseudomonas* sp. W12 [7]. It was speculated that the decrease of *Pseudomonas* spp. under consecutive monoculture might be associated with the low abundance of the clusters of defense mechanisms in a highly competitive environment. In contrast, the development of antibiotic resistance and defense mechanisms allows pathogenic or tolerant microorganisms to survive antimicrobial treatment and lead to significant deterioration of health status of plants infected. Considering the above-mentioned, the different stress response and antibiotic resistance for harmful/beneficial microbes, along with the complex plant–microbe interactions mediated by root exudates [7], might be associated with the succession of rhizosphere microbial community and the development of soil-borne diseases under monoculture.

## 4. Materials and Methods

### 4.1. Field Experiment and Soil Sampling

A common variety, *R. glutinosa* “Wen 85-5”, generally planted on a large-scale in the main production region, was selected for this study. It was generally planted in April and harvested in October of the same year, with five phenological phases: germination stage, seedling stage, root elongation stage, root expansion stage, and harvest stage. The experiment was conducted at Jiaozuo City, Henan Province (34°56′ N, 112°58′ E), known as the “geo-authentic” zone for *R. glutinosa* cultivation. It has a continental monsoon climate, an annual mean temperature of 14.3 °C and an annual mean precipitation of 552 mm [7]. A field previously cultivated with wheat was used for this experiment. The soil physico-chemical parameters were as follows: pH 7.52, soil organic matter 10.32 g·kg^−1^, available potassium 251.34 mg·kg^−1^, total potassium 7.23 g·kg^−1^_,_ available nitrogen 21.34 mg·kg^−1^, total nitrogen 0.48 g·kg^−1^, available phosphorus 47.02 mg·kg^−1^, and total phosphorus 1.36 g·kg^−1^. To keep the same soil physico-chemical parameters and climatic conditions, two different treatments were included within a single field site: (i) the one-year culture (also known as the newly planted, NP); (ii) two-year consecutive monoculture (CM). *R. glutinosa* for CM was cultivated on 15 April 2013 and reaped on 30 October 2013, and then replanted on 15 April 2014. All plots were kept fallow after the harvest (from 31 October 2013 to 14 April 2014). *R. glutinosa* for NP was cultivated on 15 April 2014. Namely, all plots (NP and CM) were planted in different years and then sampled at the same time/year to avoid the effects of climatic variation on soil microbial community. All study plots were closely adjacent with 1-m-deep separation walls that were covered with impermeable membranes to avoid the mutual influence among different treatments. Prior to planting, each plot received four fertilizers: 1.25 kg (NH_4_)_2_HPO_4_, 1.6 kg N–P–K complex fertilizer, 1.6 kg Ca(H_2_PO_4_)_2_, and 0.8 kg K_2_SO_4_. The same fertilization and water management were performed for all study plots during the entire experimental period.

On 20 September 2014, soil samples were collected from five random locations within each plot because of significant differences in plant performance between NP and CM (Figure 1). Soil samples from two different treatments were collected at the same time. As soil collected from five random locations within each plot was mixed together to make composite samples, three biological replicates were obtained for each treatment. For soil sampling, fresh plants were carefully uprooted from the soil with a forked spade and slightly shaken to remove loosely attached soil [7]. The rhizosphere soil tightly attached to tuberous roots was collected. Soil samples were sieved (2 mm mesh) and then used for soil DNA extraction.

### 4.2. DNA Extraction, Library Construction, and Metagenomic Sequencing

For each soil sample, the extraction of total soil DNA was performed using a BioFast soil Genomic DNA Extraction kit (BioFlux, Hangzhou, China) following the manufacturer’s instructions [4]. The quality of soil DNA was checked using 1% agarose gels and a NanoPhotometer spectrophotometer (IMPLEN, Westlake Village, CA, USA). The concentration of soil DNA was determined through a Qubit^®^ dsDNA Assay Kit in Qubit 2.0 Flurometer (Life Technologies, Carlsbad, CA, USA).

For the DNA sample preparations, a total of 1 μg DNA per sample was used as input material. Sequencing libraries were generated using NEBNext^®^ Ultra™ DNA Library Prep Kit for Illumina (NEB, Ipswich, MA, USA) following manufacturer’s recommendations and index codes were added to attribute sequences to each sample [48]. In brief, the DNA sample was fragmented by sonication to a size of approximately 300 bp, then DNA fragments were end-polished, A-tailed, and ligated with the full-length adaptor for Illumina sequencing with further polymerase chain reaction (PCR) amplification. Finally, PCR products were purified (AMPure XP system, Beckman Coulter, Brea, CA, USA) and libraries were analyzed for size distribution by Agilent2100 Bioanalyzer (Agilent Technologies, Palo Alto, CA, USA) and quantified using real-time PCR [48].

The clustering of the index-coded samples was performed on a cBot Cluster Generation System (Illumina, San Diego, CA, USA) according to the manufacturer’s instructions. After cluster generation, the library preparations were sequenced on an Illumina HiSeq X Ten platform at NOVOgene Company (Beijing, China) and 150 bp paired-end reads were generated [48]. The sequence data have been submitted to NCBI Short Read Archive (SRA) under BioProject PRJNA445836, accession number SRP136591.

### 4.3. Sequence Quality Control and Assembly

To improve the reliability of data processing, raw data from HiSeq X Ten platform were trimmed to obtain the high-quality clean reads (clean data) according to the quality control parameters using low-quality base (score ≤ 38) 40 bp, ambiguous base (N) 10 bp, and overlap with adapter sequences 15 bp as cutoff. The adapters and sequences from the host plant were stripped as well. Clean data per sample was then assembled to produce Scaffolds using SOAPdenovo software [49] with parameters “-d 1, -M 3, -R, -u, -F”. Scaffolds were divided into Scaftigs without gaps (N). Scaftigs with a length shorter than 500 bp were removed for further gene prediction and annotation.

### 4.4. Gene Prediction, Phylogenetic Comparison and Functional Annotation

Scaftigs (≥500 bp) from each sample were used for open reading frame (ORF) prediction through MetaGeneMark [50] with the parameters “gmhmmp, -a, -d, -f G, -p 1, -m MetaGeneMark_v1.mod”. The predicted ORFs (≥100 nt) redundancy was removed using Cluster Database at High Identity with Tolerance (CD-HIT) [51] program with parameters “-c 0.95, -G 0, -aS 0.9, -g 1, -d 0” in order to obtain an initial non-redundant gene catalogue (nrGC). The reads from each sample were realigned to the nrGC using SoapAligner (Shenzhen, Guangdong, China) [52] with the parameters “-m 200, -x 400, identity ≥ 95%”. The read number of each non-redundant gene (namely, Unigenes) successfully mapped to the initial nrGC was used to calculate the abundance. Among them, the genes that contain ≤2 sequences in all six soil samples were removed for further analysis. We calculated the relative abundance of a gene by counting the number of reads that align to the gene normalizing by the total number of reads aligned to any contig and the gene length [53]. Then, the relative abundance of each gene is multiplied by the maximum of reads number mapping to the non-redundant gene catalogue among six soil samples to obtain homogenized reads number. Core/pan and Venn diagram analyses were carried out to detect the exclusive and shared genes among different soil samples.

The alignment of sequencing reads against the NCBI microNR database (including bacteria, fungi, archaea, and viruses, version: 19 October 2014) was then conducted using DIAMOND [54] (blastp, e value ≤ 1 × 10^−5^) to obtain the phylogenetic information of Unigenes. The phylogenetic information of each gene was determined using the lowest common ancestor-based algorithm (LCA) implemented in MEGAN (version 4, Tübingen, Baden-Württemberg, Germany) [55]. The relative abundance for each taxonomical rank was calculated by summing up the relative abundance of all its members [53]. Kyoto Encyclopedia of Genes and Genomes (KEGG) annotation and cluster of orthologous groups of proteins (COG) of each gene were performed using DIAMOND (blastp, e value ≤ 1 × 10^−5^) against KEGG database [56] and Evolutionary genealogy of genes: Non-supervised Orthologous Groups (eggNOG) database (version: 4.1) [57], respectively. We calculated the relative abundance per feature by summing up the abundances of genes annotated to a feature [53]. The relative abundance of a feature derived from a specific taxon was calculated by summing up the relative abundances of all its members assigned to this feature.

### 4.5. Statistical Analyses

The tuber weight of the one-year planted and two-year monocultured *R. glutinosa* was compared using Student’s *t*-test. Metastats method (non-parametric permutation test, *p* < 0.05, *n* = 3) was applied to identify differentially abundant features in metagenomics sequence datasets [58]. *p*-Values were adjusted with false discovery rate (FDR) [58] for multiple testing and are denoted *q*-values. A *q*-value threshold of 0.2 (FDR < 20%) were used for significance to minimize missing true discoveries [59,60,61]. Principal component analysis (PCA) and unweighted pair-group method with arithmetic mean (UPGMA) clustering based on Bray–Curtis distances were performed to investigate beta-diversity patterns. The heat maps for microbial taxa and functional features were generated using R software version 3.1.3 and color-coded by row z-scores.

## 5. Conclusions

**Figure 6:**
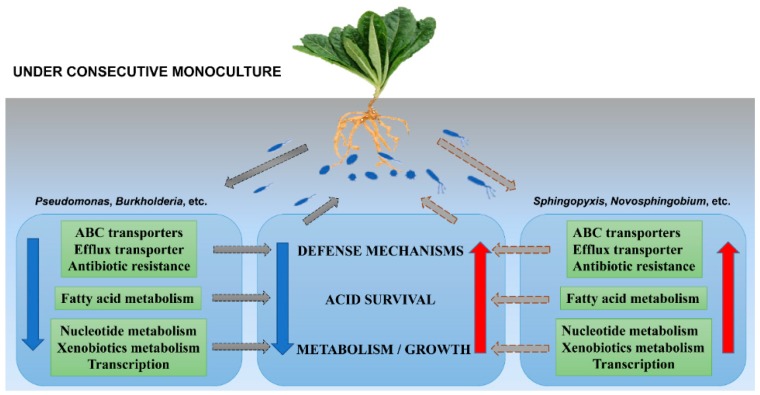
Model illustrating the proposed microbial phylogenetic and functional responses to *R. glutinosa* consecutive monoculture. Dashed grey arrows represent the process flow sequence. Blue solid arrows represent the down-regulation of functional potentials in several microbes under monoculture and red solid arrows represent the up-regulation of functional potentials in several microbes under monoculture.

Our results demonstrated that consecutive monoculture of *R. glutinosa* altered the structure and composition of microbial communities in the rhizosphere, leading to relatively fewer beneficial microorganisms (i.e., *Lysobacter*, *Pseudomonas*, *Azotobacter*, *Burkholderia*). Furthermore, whole-genome shotgun sequencing enabled association of the functional genes to the phylogenetic diversity of the complex rhizosphere microbial communities, and offered meaningful insights into community functional responses to consecutive monoculture (Figure 6). Further studies will be performed to investigate functional gene expression with metatranscriptomics, while metabolites will be analyzed through a metabolomics approach. In addition, further work on the endophytic microbial community in *R. glutinosa* monoculture regime is needed.

## Supplementary Materials

Supplementary materials can be found at http://www.mdpi.com/1422-0067/19/8/2394/s1.

## Abbreviations

CM	two-year consecutively monocultured soil
NP	newly planted soil (one-year cultured soil)
T-RFLP	terminal restriction fragment length polymorphism
PLFA	phospholipid fatty acid
ORF	open reading frame
KEGG	Kyoto Encyclopedia of Genes and Genomes
eggNOG	Evolutionary genealogy of genes: Non-supervised Orthologous Groups
KO	KEGG ortholog group
PCA	principal component analysis
PC1	the first principal component
PC2	the second principal component
UPGMA	unweighted pair-group method with arithmetic mean
SRA	Short Read Archive
nrGC	non-redundant gene catalogue
LCA	lowest common ancestor-based algorithm
COG	cluster of orthologous groups of proteins
FDR	false discovery rate
