# Supplementary material for: Comparative Metagenomic Analysis of Rhizosphere Microbial Community Composition and Functional Potentials under Rehmannia glutinosa Consecutive Monoculture
Source: Int J Mol Sci. 2018 Aug 14;19(8):2394. doi: 10.3390/ijms19082394 (PMC6121535; doi:10.3390/ijms19082394)
Supplement: Supplementary file 1 [file ijms-19-02394-s001.pdf]

Supplementary Materials: Comparative Metagenomic Analysis of Rhizosphere Microbial Community Composition and Functional Potentials under *Rehmannia glutinosa* Consecutive Monoculture

Linkun Wu, Juanying Wang, Hongmiao Wu, Jun Chen, Zhigang Xiao, Xianjin Qin, Zhongyi Zhang and Wenxiong Lin

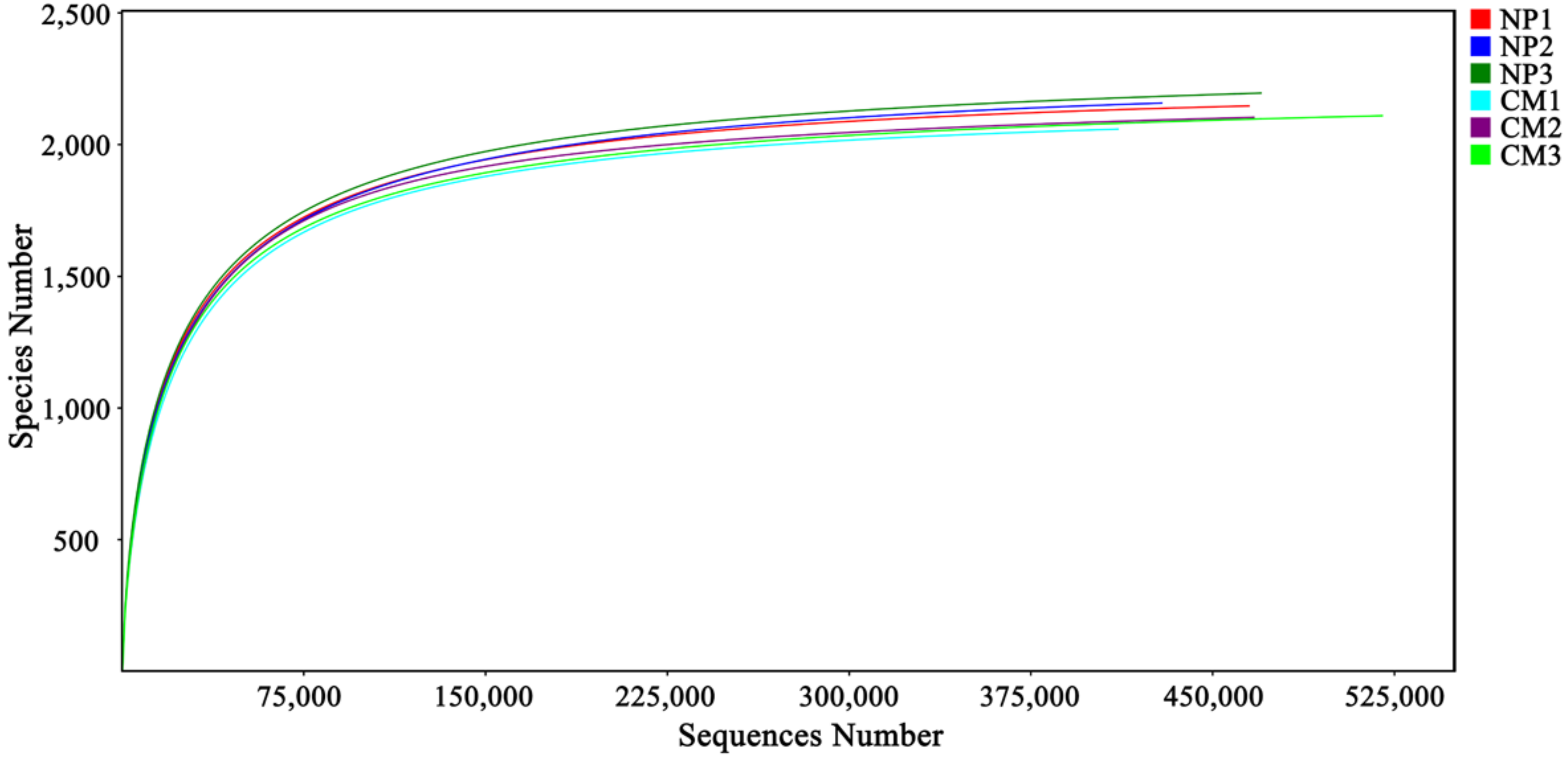

Figure S1. Rarefaction curves generated for the species level based on metagenomics data. NP and CM represent the one-year cultured soil and two-year consecutively monocultured soil, respectively.

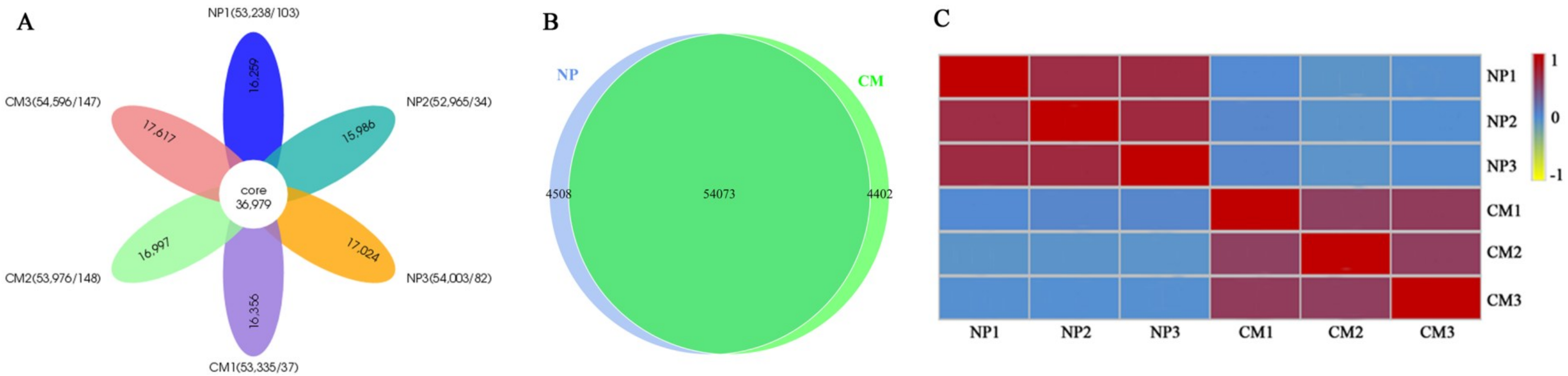

Figure S2. Core/pan analysis (A), Venn diagram analysis (B) and correlation analysis among different samples (C) based on metagenomics data. A: The number in the core represents the number of genes shared among six soil samples. The numbers in a parenthesis represent the total number of genes in each sample and the number of exclusive genes found in each sample, respectively. The number in a petal represents the difference between the total number of genes in each sample and the number of genes shared among six soil samples. B: The overlap represents the number of genes shared by two different treatments, and the two sides represent the number of exclusive genes in each treatment. C: The value in the legend represents Spearman's rank correlation coefficient calculated from the normalized abundance of each gene, with a value near 1 indicating 100% similarity between different groups. NP and CM represent the one-year cultured soil and two-year consecutively monocultured soil, respectively.

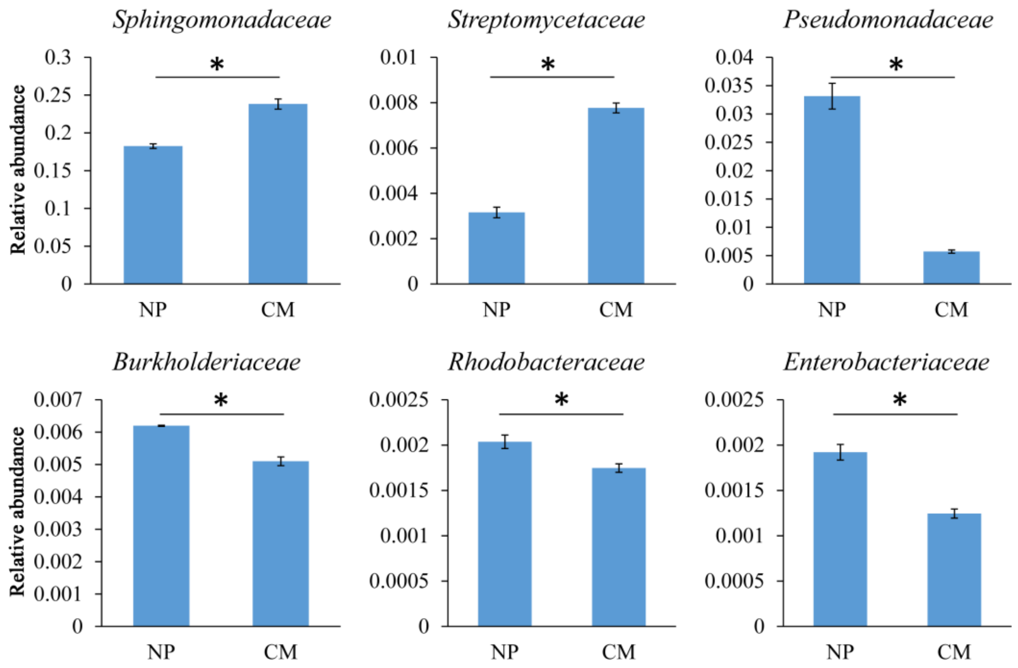

Figure S3. Relative abundances of the six predominant bacterial families showing significant difference (*, $q < 0.05$) between the one-year and two-year monocultured plots. NP and CM represent the one-year cultured soil and two-year consecutively monocultured soil, respectively.

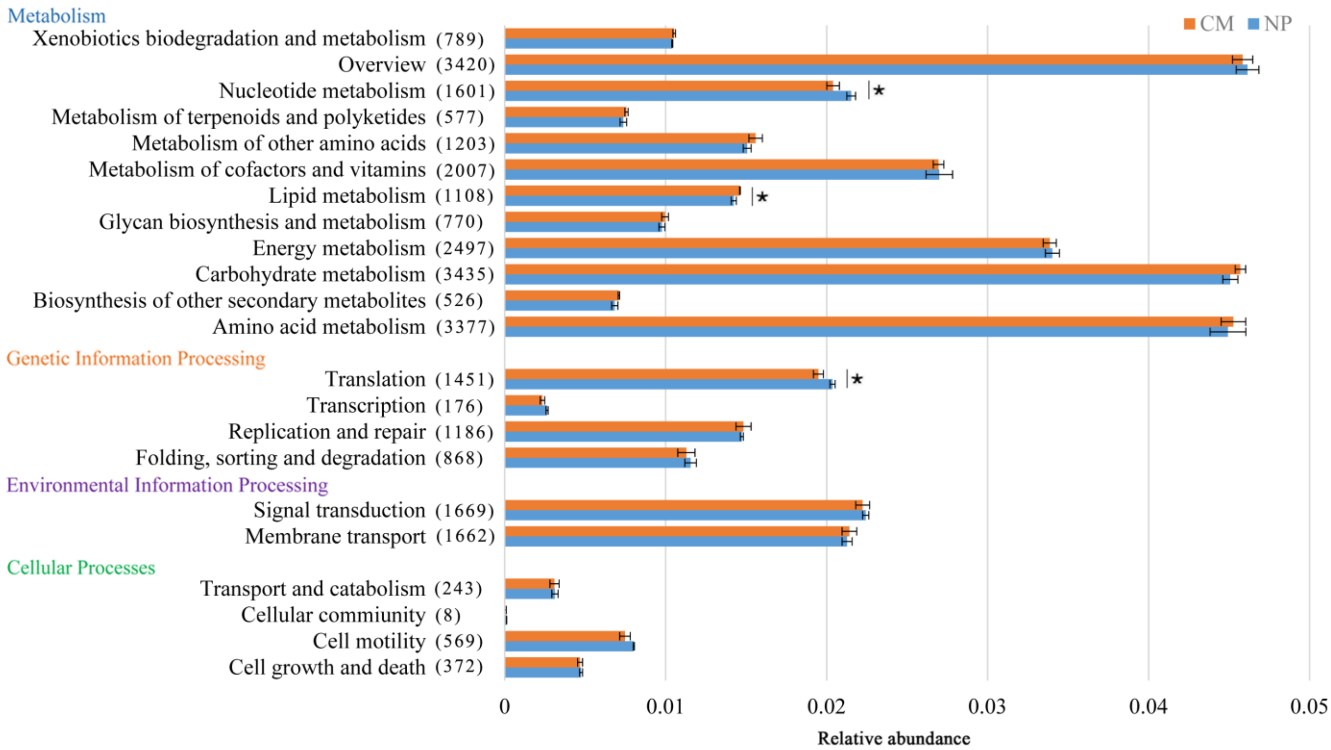

Figure S4. Relative abundances of the genes annotated to each functional class/metabolic pathway on KEGG database. The numbers in the parentheses represent the total numbers of genes annotated to each functional class/metabolic pathway from NP and CM. NP and CM represent the one-year cultured soil and two-year consecutively monocultured soil, respectively. The Unigenes assigned to metabolic pathways of 'Organismal Systems', 'Human Diseases' on KEGG database were grouped into 'Others' group, not shown here. * represents significant differences for KEGG pathways between two different treatments ($q < 0.05$).

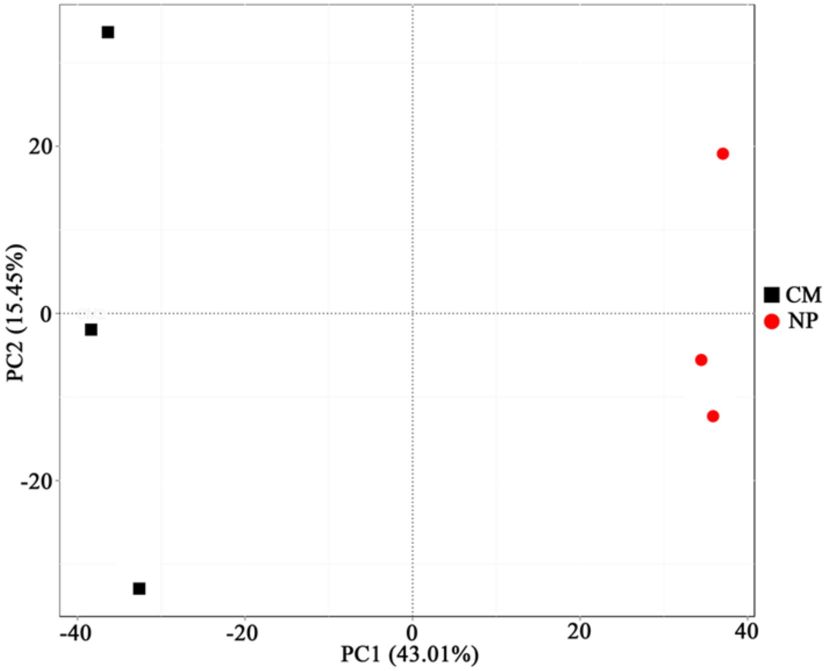

Figure S5. Principal component analysis (PCA) based on the relative abundances of KEGG ortholog (KO) groups for two different treatments. NP and CM represent the one-year cultured soil and two-year consecutively monocultured soil, respectively.

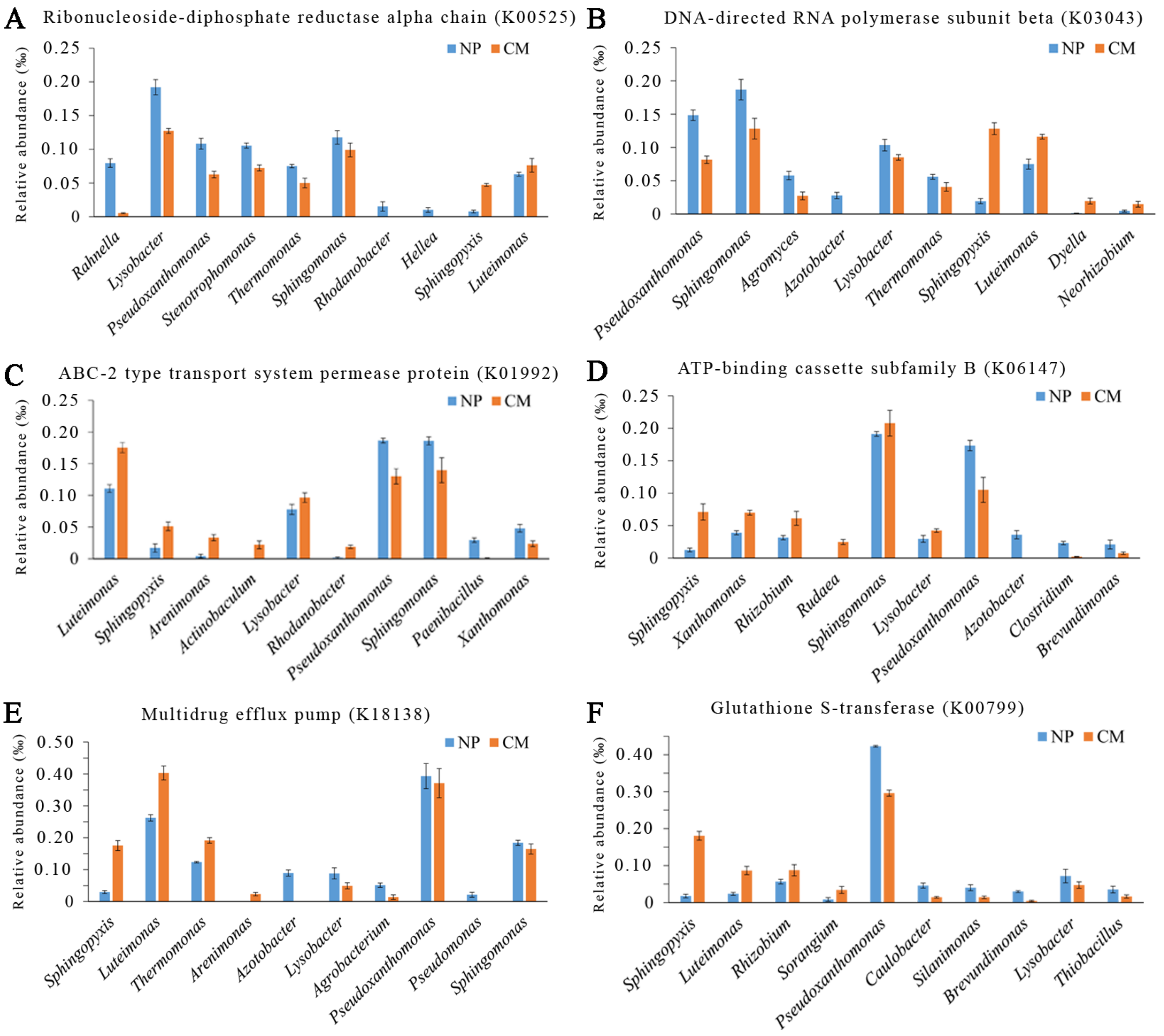

Figure S6. The top 10 genera contributing to the dissimilarity of K00525 (A), K03043 (B), K01992 (C), K06147 (D), K18138 (E) and K00799 (F) between two different treatments. NP and CM represent the one-year cultured soil and two-year consecutively monocultured soil, respectively.

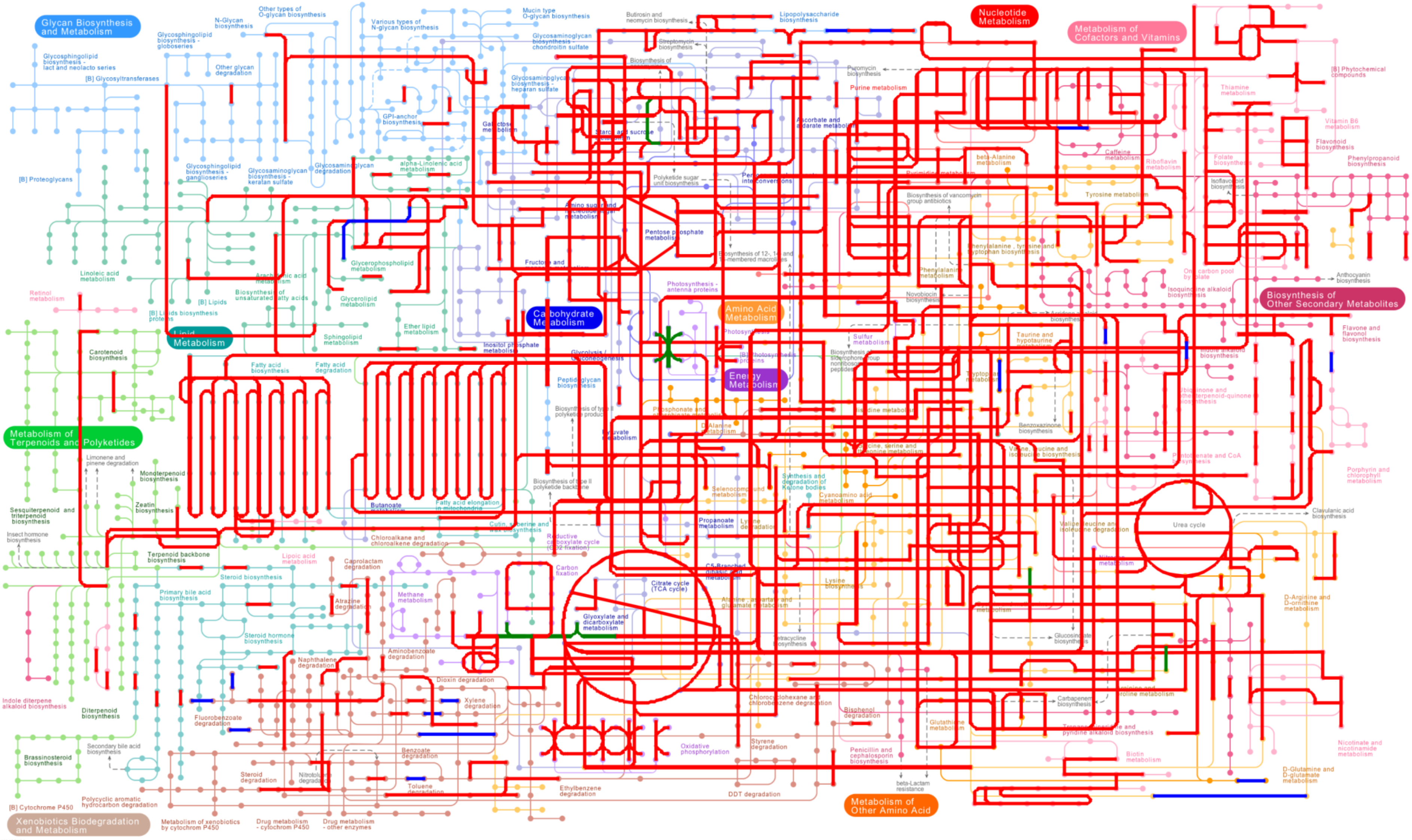

Figure S7. Annotated KEGG metabolic pathways based on the metagenomic data from NP and CM. The bold red lines represent the biochemical reactions detected in both NP and CM. The bold blue and green lines represent the biochemical reactions only detected in NP and CM, respectively. NP and CM represent the one-year cultured soil and two-year consecutively monocultured soil, respectively.

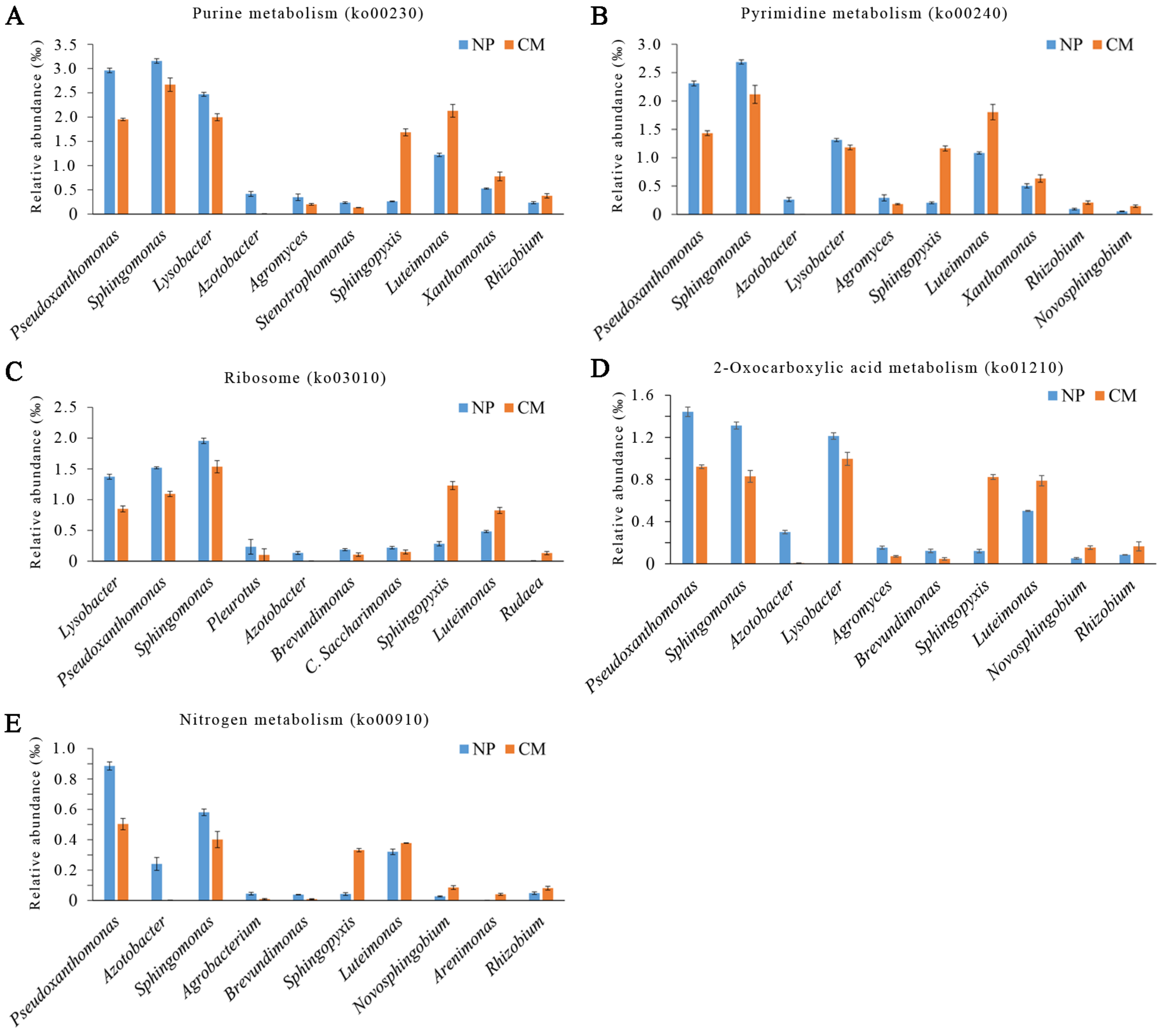

Figure S8. The top 10 genera contributing to the dissimilarity of ko00230 (A), ko00240 (B), ko03010 (C), ko01210 (D) and ko00910 (E) between two different treatments. NP and CM represent the one-year cultured soil and two-year consecutively monocultured soil, respectively.

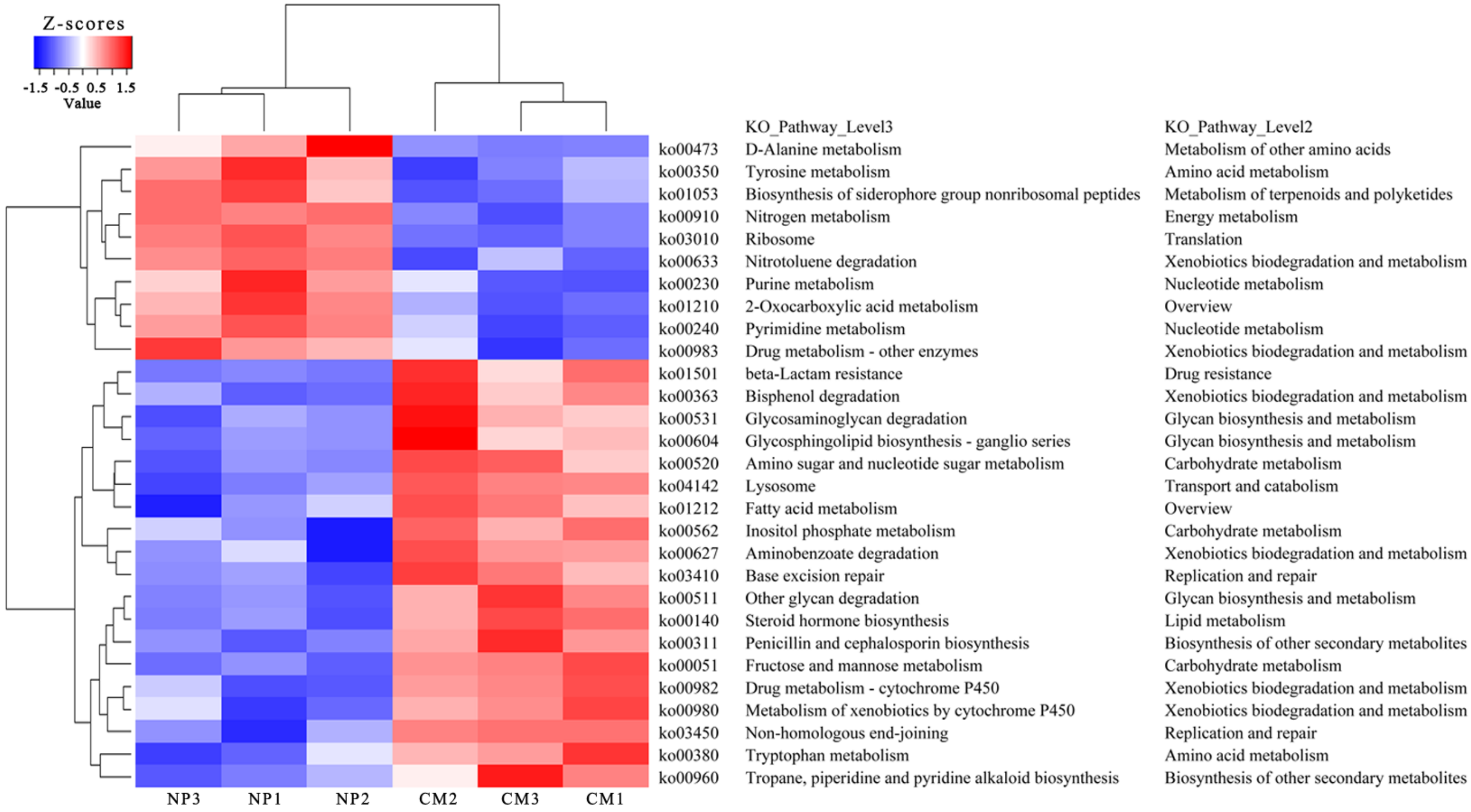

Figure S9. Heat map analysis of all significantly ($q < 0.12$) differential KEGG pathways (level 3) using normalized abundance between two different treatments. Heat map is color-coded based on row z-scores. A metabolic pathway (ko01501) assigned to 'Human Diseases' on KEGG database is included here. NP and CM represent the one-year cultured soil and two-year consecutively monocultured soil, respectively.

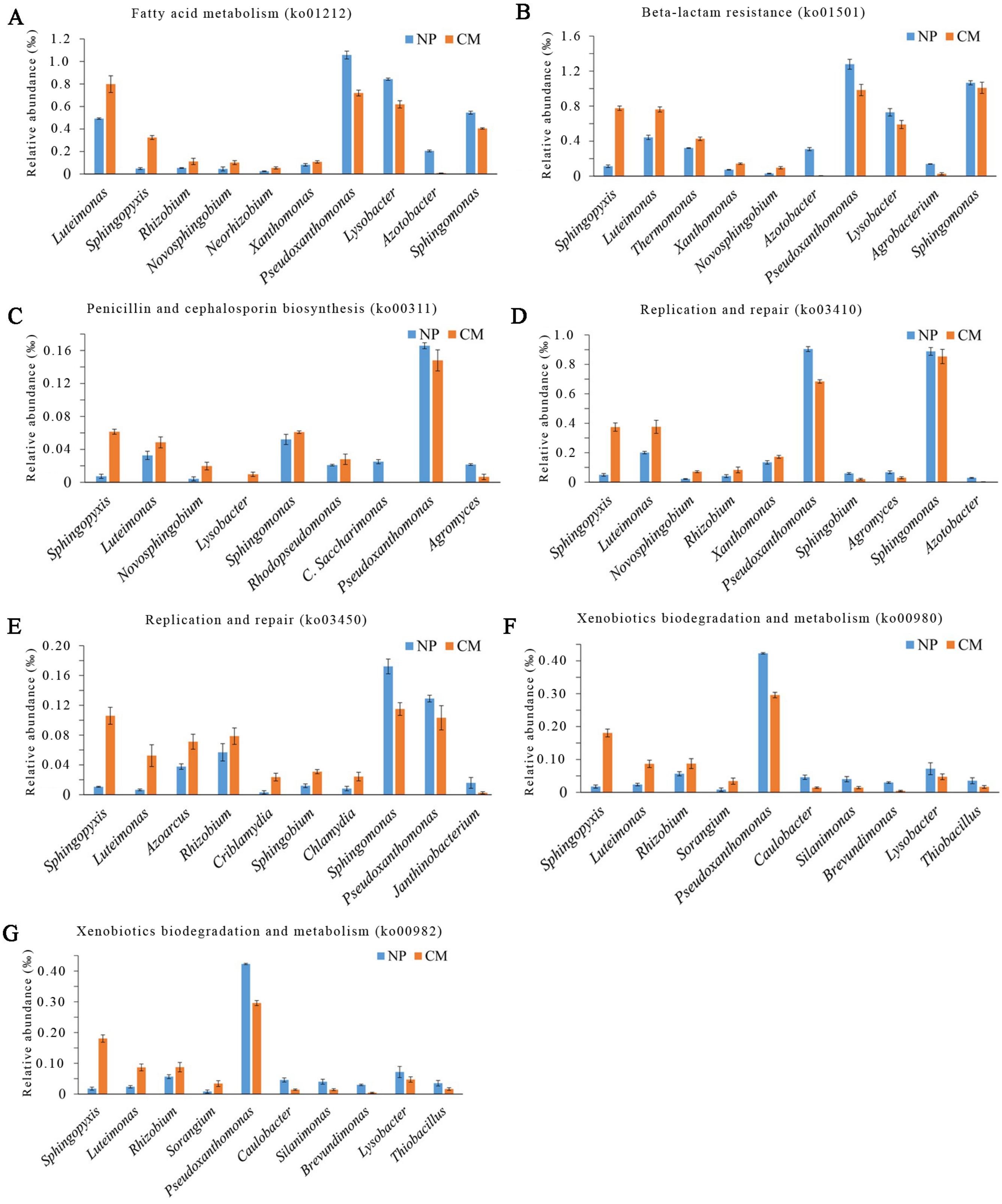

Figure S10. The top 10 genera contributing to the dissimilarity of ko01212 (A), ko01501 (B), ko00311 (C), ko03410 (D), ko03450 (E), ko00980 (F) and ko00982 (G) between two different treatments. NP and CM represent the one-year cultured soil and two-year consecutively monocultured soil, respectively.

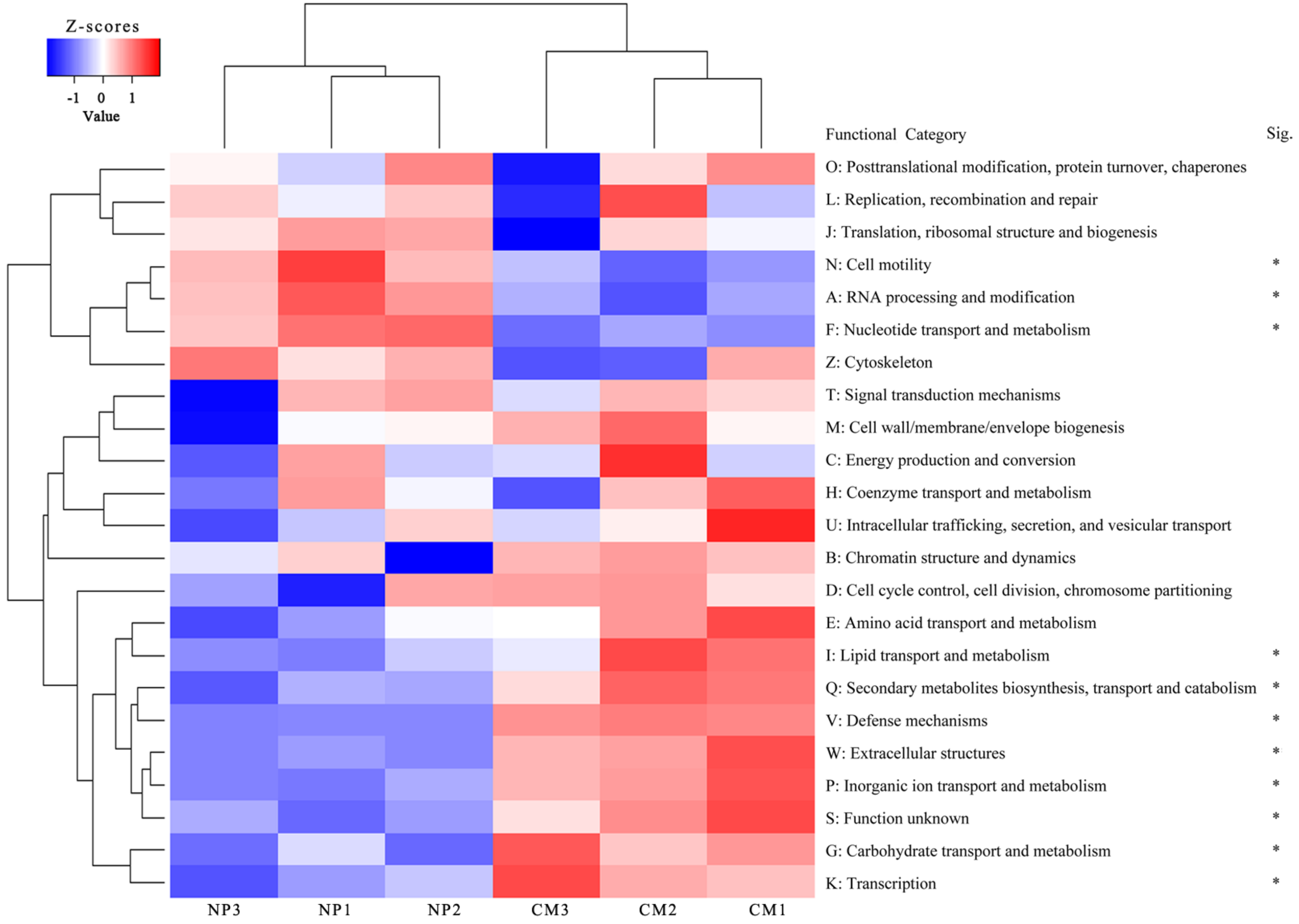

Figure S11. Heat map analysis of eggNOG functional category (level 1) using normalized abundance between two different treatments. Heat map is color-coded based on row z-scores. NP and CM represent the one-year cultured soil and two-year consecutively monocultured soil, respectively. Sig. represents significant differences between two different treatments (*, $q < 0.05$).

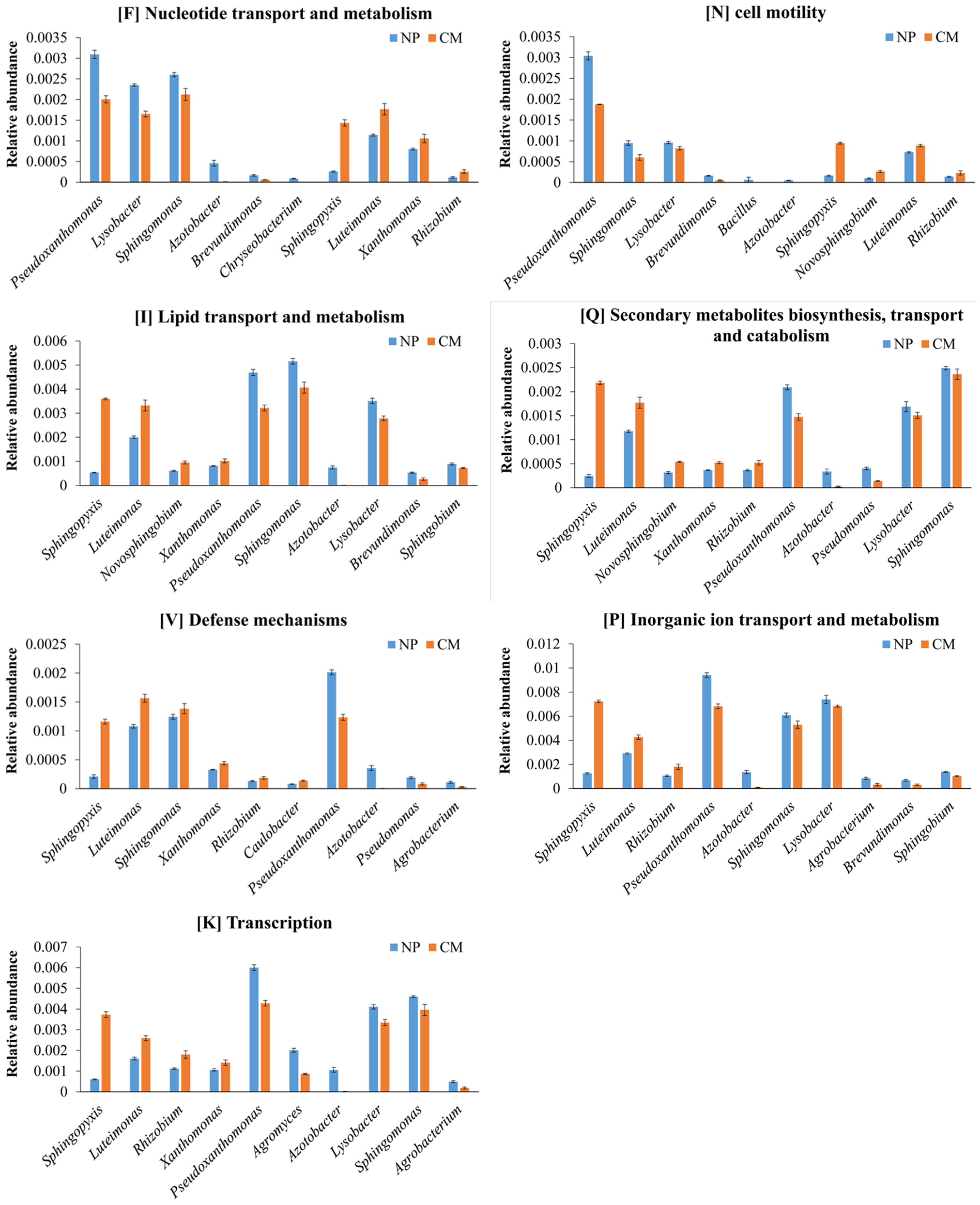

Figure S12. The top 10 genera contributing to the dissimilarity of eggNOG functional clusters between two different treatments. NP and CM represent the one-year cultured soil and two-year consecutively monocultured soil, respectively.

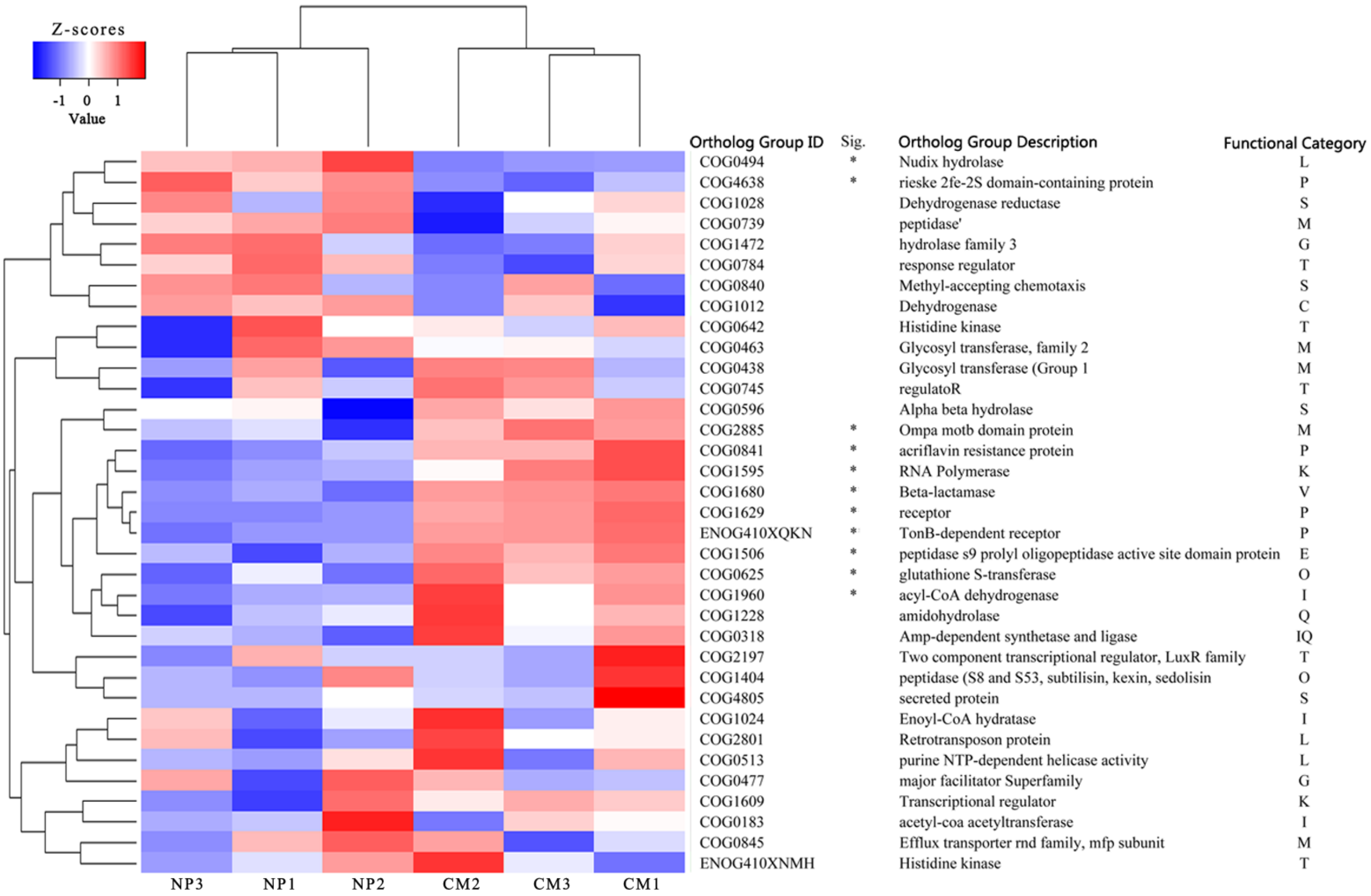

Figure S13. Heat map analysis of the top 35 most abundant eggNOG orthologous groups in at least one soil sample using normalized abundance. Heat map is color-coded based on row z-scores. NP and CM represent the one-year cultured soil and two-year consecutively monocultured soil, respectively. Sig. represents significant differences for eggNOG orthologous groups between two different treatments (*, $q < 0.06$). Abbreviation codes for each functional category were presented in Figure S11.

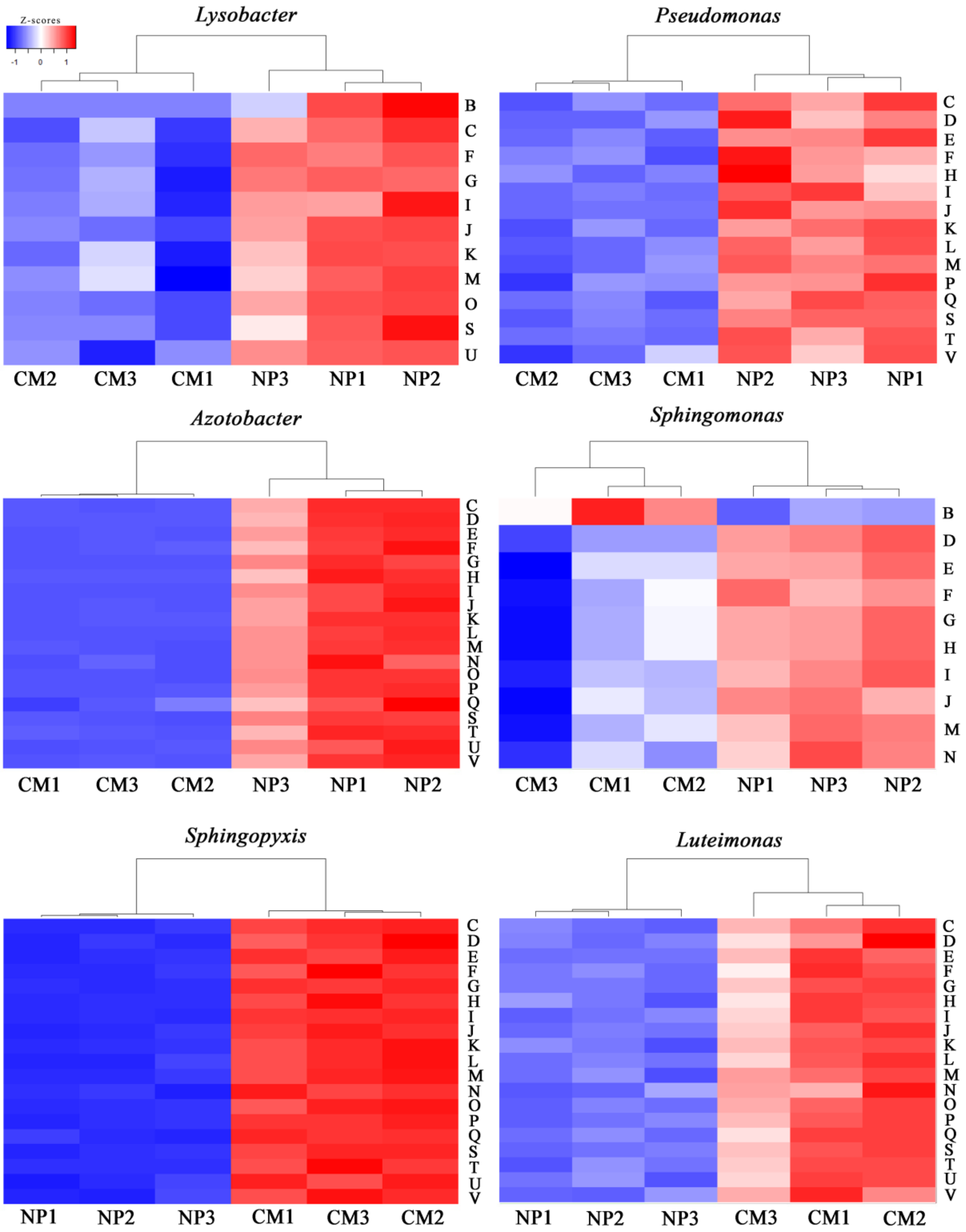

Figure S14. Heat map analysis of differential eggNOG orthologous groups of specific microbes using normalized abundance between two different treatments. Heat map is color-coded based on row z-scores. NP and CM represent the one-year cultured soil and two-year consecutively monocultured soil, respectively. Abbreviation codes for each functional category were presented in Figure S11.

Table S1. Summary for metagenomic sequencing, assembly and annotation.

Category	NP1	NP2	NP3	CM1	CM2	CM3	In total
Scaftigs total length (bp)	12,121,751	10,521,208	10,331,527	9,252,009	12,891,147	14,157,689	69,275,331
Scaftigs number	14,014	12,426	11,875	11,865	16,488	15,344	82,012
Scaftigs average length (bp)	865	847	870	780	782	923	845
Scaftigs N50 length (bp)	847	830	855	761	756	923	829
Scaftigs N90 length (bp)	542	538	541	533	535	549	540
ORFs number	21,369	18,702	18,234	16,770	23,385	23,637	71,246#
Integrity:all	4,742(22.19%)	3,993(21.35%)	4,192(22.99%)	2,863(17.07%)	3,955(16.91%)	5,331(22.55%)	15,672#(22.00%)
Integrity:start	7,717(36.11%)	6,891(36.85%)	6,826(37.44%)	6,421(38.29%)	8,930(38.19%)	8,543(36.14%)	24,487#(34.37%)
Integrity:end	5,442(25.47%)	4,725(25.26%)	4,419(24.23%)	4,377(26.1%)	6,076(25.98%)	6,031(25.52%)	17,216#(24.16%)
Integrity:none	3,468(16.23%)	3,093(16.54%)	2,797(15.34%)	3,109(18.54%)	4,424(18.92%)	3,732(15.79%)	13,871#(19.47%)
ORFs total length (Mbp)	10.72	9.3	9.08	8.18	11.49	12.52	38.32*
ORFs average length (bp)	501.50	497.18	498.21	488.03	491.39	529.68	537.82*
ORFs GC percent (%)	64.93	65	63.88	64.4	65.09	63.38	64.28*

NP and CM represent the one-year cultured soil and two-year consecutively monocultured soil, respectively. The numbers followed by the treatments represent the three replicates.

Scaftigs N50 length, the length of the smallest scaftigs in the set of largest scaftigs that have a combined length that represents at least 50% of the assembly.

Scaftigs N90 length, the length of the smallest scaftigs in the set of largest scaftigs that have a combined length that represents at least 90% of the assembly.

# represents the number of non-redundant ORFs; * represents the total length, average length and GC percent of non-redundant ORFs.

Integrity:all, the complete ORFs containing both initiation codon and termination codon.

Integrity:start, the predicted ORFs containing only initiation codon.

Integrity:end, the predicted ORFs containing only termination codon.

Integrity:none, the predicted ORFs without initiation codon and termination codon.
